# Climate change triggered planktonic cyanobacterial blooms in a regulated temperate river

**DOI:** 10.1038/s41598-024-66586-w

**Published:** 2024-07-15

**Authors:** Julia Kleinteich, Marieke A. Frassl, Manoj Schulz, Helmut Fischer

**Affiliations:** Federal Institute of Hydrology (BfG), Am Mainzer Tor 1, 56068 Koblenz, Germany

**Keywords:** Harmful algae bloom, Eutrophication, Discharge, Water temperature, *Microcystis*, Phytoplankton, Freshwater ecology, Freshwater ecology, Microbial ecology, Limnology, Water microbiology, Climate sciences

## Abstract

Harmful algae blooms are a rare phenomenon in rivers but seem to increase with climate change and river regulation. To understand the controlling factors of cyanobacteria blooms that occurred between 2017 and 2020 over long stretches (> 250 km) of the regulated Moselle River in Western Europe, we measured physico-chemical and biological variables and compared those with a long-term dataset (1997–2016). Cyanobacteria (*Microcystis*) dominated the phytoplankton community in the late summers of 2017–2020 (cyano-period) with up to 110 µg Chlorophyll-a/L, but had not been observed in the river in the previous 20 years. From June to September, the average discharge in the Moselle was reduced to 69–76% and water temperature was 0.9–1.8 °C higher compared to the reference period. Nitrogen (N), phosphorus (P) and silica (Si) declined since 1997, albeit total nutrient concentrations remained above limiting conditions in the study period. Cyanobacterial blooms correlated best with low discharge, high water temperature and low nitrate. We conclude that the recent cyanobacteria blooms have been caused by dry and warm weather resulting in low flow conditions and warm water temperature in the regulated Moselle. Under current climate projections, the Moselle may serve as an example for the future of regulated temperate rivers.

## Introduction

Excessive growth of phytoplankton has caused a number of harmful events in European waters in recent years and indicates that climate change may bring back phenomena from a eutrophic past. Particularly, mass occurrences of toxic cyanobacteria and other harmful algae are an unwanted phenomenon. Harmful algae blooms (HABs) not only pose a threat to humans and livestock, they also increase economic expenses for water treatment or damage fish and marine shellfish^1^. The seemingly global increase in harmful algae blooms, and especially those caused by cyanobacteria, is generally attributed to the effects of eutrophication and climate change^2^.

Several factors supporting cyanobacterial blooms, like nutrient enrichment or rising water temperatures act similar across aquatic ecosystems. However, there is a knowledge gap regarding specific factors leading to harmful algae blooms in rivers, like the variability and change in flow^3^. This is reflected in reviews about cyanobacteria blooms, where large rivers are much less prominent than marine ecosystems and lakes^4–6^. A review that mentions hydrological changes as a potential cause for the spread of cyanobacteria blooms in rivers mainly provides examples from river deltas and from less inland rivers^7^. Riverine cyanobacteria blooms have been observed in tropical and subtropical regions, such as Australia^8^, but also rivers of temperate regions in North America or in Europe have been affected occasionally^9,10^. Cyanobacteria blooms in rivers seem to occur under low flow conditions^3,8,9^ in combination with low turbulence and turbidity^8^. Sufficient discharge and flow velocity on the other hand can prevent mass development of cyanobacteria even under high nutrient conditions^8^.

Climate change scenarios for temperate regions like western and central Europe project an extension and intensification of dry summer periods with higher temperatures and increasing droughts^11^. In Western Europe, the years from 2015 to 2019 were characterized by extended drought conditions, which led to a long-lasting decrease in discharge in many European rivers^12^. Reduced discharge can lead to a lower flow velocity and to an increased residence time. These hydrological factors in combination with elevated water temperature and increased solar radiation during warm and dry weather periods may promote the growth of cyanobacteria in rivers^6^. Regulated rivers may be especially sensitive to such consequences of climate change. In those rivers, dams artificially regulate the water level to serve multiple purposes such as flood control, hydropower generation or shipping^13^. These engineered in-stream structures extend residence times especially under low discharge conditions^14^. Moreover, they enhance sediment deposition and nutrient retention^15^, which may enhance algal or cyanobacterial growth^16–18^.

In the Moselle, a regulated river between France, Luxemburg and Germany, cyanobacterial blooms have occurred since 2017 and have stretched over more than 250 river km. The Moselle is regulated by 28 impoundments that have been constructed or enlarged in the 1960s for navigation and hydroelectricity. The Moselle fulfills a number of ecosystem services. The Moselle landscape is a touristic region and the river is popular for cruise shipping, cycling and wine production. It also serves as drinking water resource from river bank filtration. Though no fish kills or human health effects have yet been reported in the Moselle, the sudden occurrence of cyanobacterial blooms is a worrying development for local residents and authorities since such blooms had not been reported before in the river and clearly show a deterioration of the river’s water quality. In this study we hypothesize that the growth of cyanobacteria in the regulated Moselle in the years of 2017–2020 was driven by environmental factors resulting from warm and dry summer periods in Western Europe. We analyzed a long-term dataset and compared the cyanobacteria growth period from 2017 till 2020 with the 20 years before cyanobacteria occurrence (1997–2016) as a reference and identified drivers for the occurrence of cyanobacteria in the Moselle.

## Results

**Figure 1:**
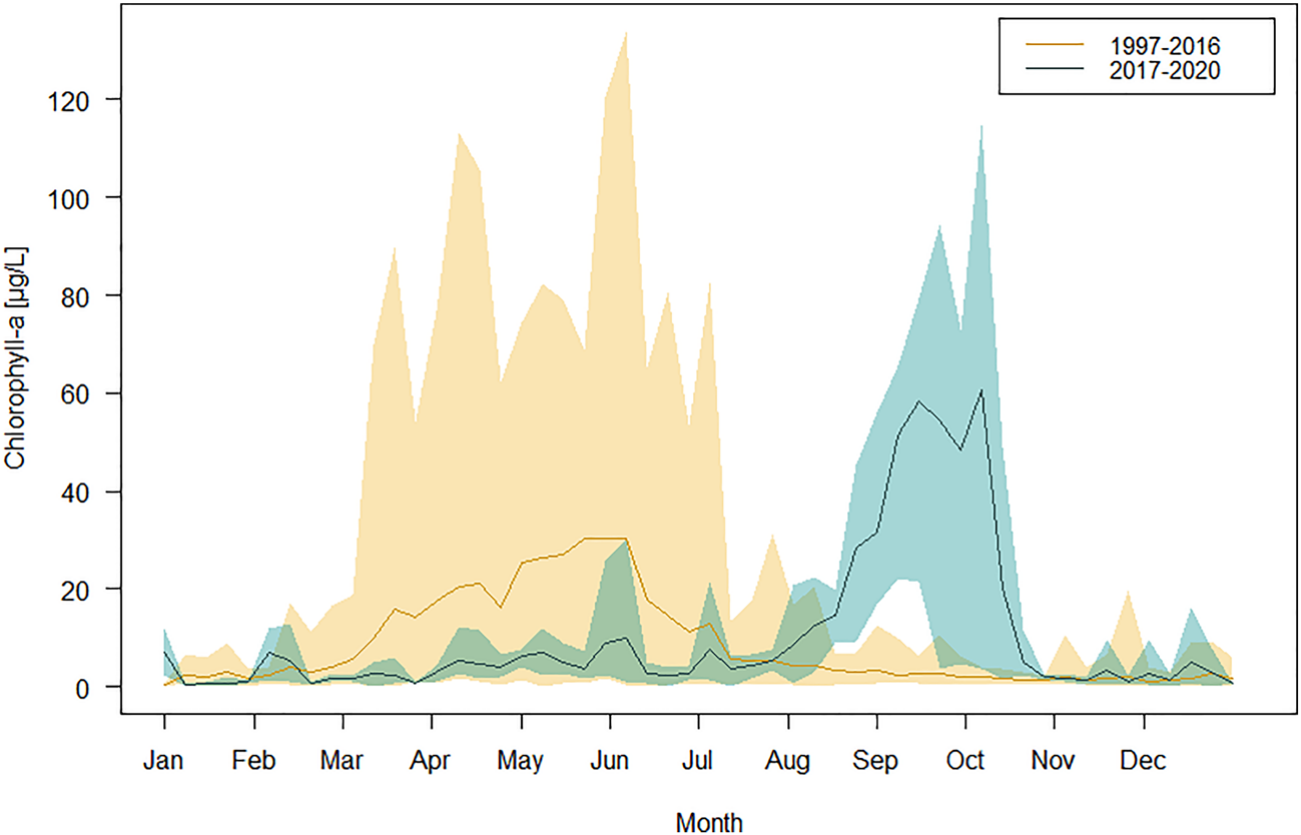
Annual chlorophyll-a (chl-a) dynamics in the Moselle River at Koblenz. Weekly mean (line) and long-term range (shaded) in a seasonal cycle for the 20-year period before cyanobacteria occurrence (1997–2016, yellow) and the years 2017–2020 (blue-green) when *Microcystis aeruginosa* formed extensive cyanobacterial blooms between August and October. Please note that spring chlorophyll was dominated by diatoms (Fig. S2) and concentrations declined in the years 2013–2016 before the occurrence of cyanobacteria (for individual chlorophyll levels per year see Fig. 3).

**Figure 2:**
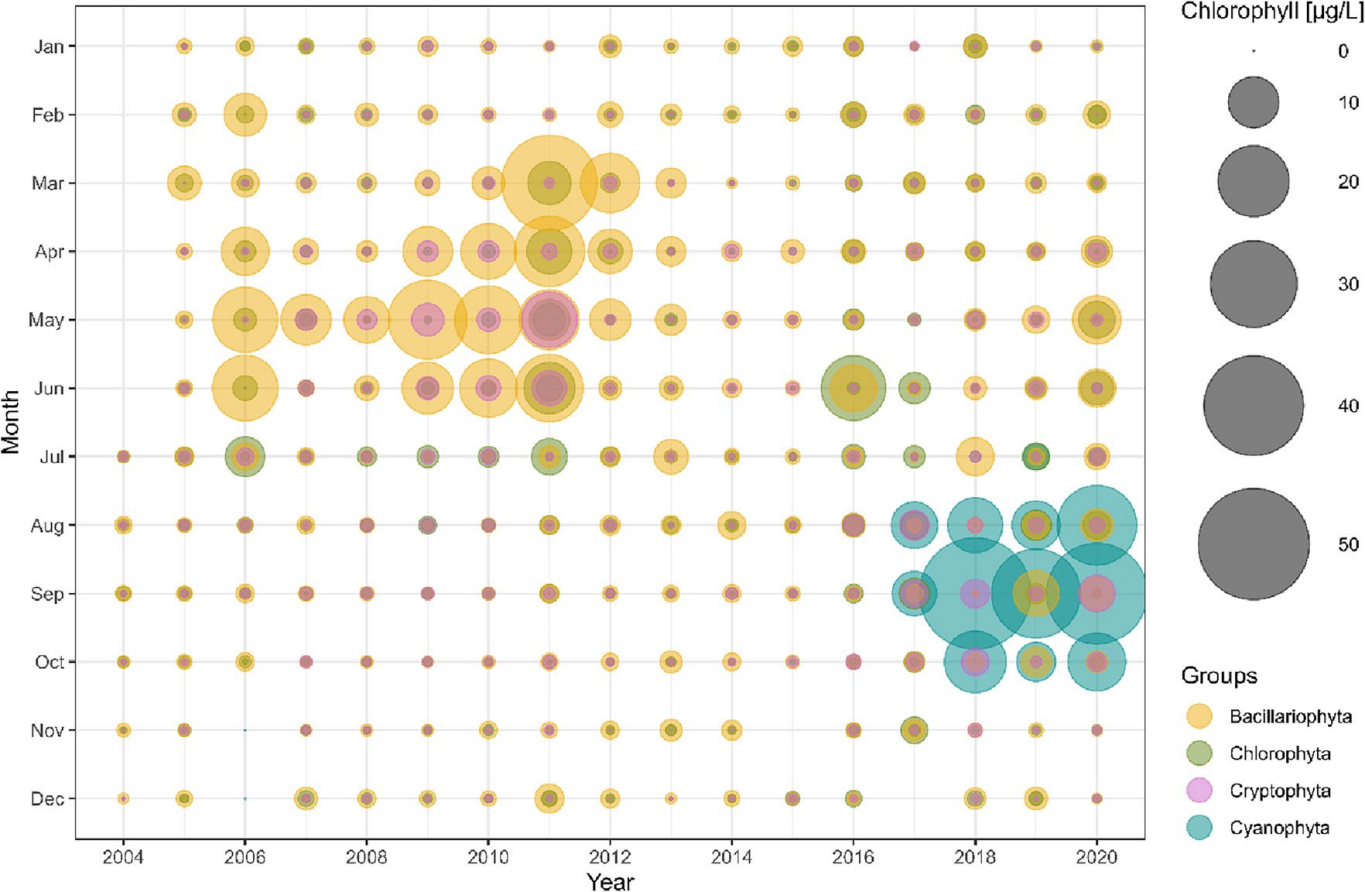
Chlorophyll-a concentration [µg/L] based on fluorescence measurements. Colors reflect major phytoplankton groups diatoms (Bacillariophyta), cyanobacteria (Cyanophyta), green algae (Chlorophyta) and cryptophytes (Cryprophyta). The size of the bubble reflects chl-*a* concentration in each group. Data are based on weekly fluorescence measurements and displayed as monthly average between 2004 and 2020. No fluorescence data are available before July 2004. The decline of the diatom spring bloom after 2012 is visible as is the onset of cyanobacteria blooms in 2017.

Summer blooms dominated by *Microcystis aeruginosa* first occurred in the Moselle in 2017 and recurred in all consecutive years during the study period (Figs. 1,2, and S2). The onset and duration of the blooms were remarkably similar in all four consecutive years: First colonies of *Microcystis* were usually observed by the end of July, while the blooms established in mid-August and reached maximum levels of up to 110 µg/L chl-*a* in September, before they declined in October. This pattern was only interrupted in 2017 when a high-discharge event in September washed out the bloom.

Cyanobacteria had not been observed in the Moselle in high abundances until the year 2017. In the 20 years reference period (1997–2016), the Moselle River at Koblenz was characterized by an annual phytoplankton bloom in spring. This spring bloom was dominated by diatoms and accompanied by minor abundances of green algae and cryptophytes (Figs. 1, 2, and S2). Spring blooms typically reached maximum chlorophyll-*a* (chl-*a*) concentrations between 50 and 130 µg/L in the months between March and June and were followed by summer periods with relatively low chl-*a* levels between 0 and 20 µg/L. The decrease of the diatom spring blooms in maximum chl-*a* concentration (up to 0–30 µg/L), and in duration, had started in 2012/13, five years before the onset of cyanobacterial blooms and coincided with the occurrence of the quagga mussel in the river^19^.

During cyanobacterial blooms, concentrations of microcystin (MC), the most common freshwater cyanobacterial toxin, were highest in August (median 0.17 µg/L) and September (median 0.11 µg/L) during the peak of the bloom (Fig. S3). Concentrations were far below the recreational water guideline by the WHO of 24 µg/L and only one sample in September 2020 exceeded the drinking water guideline of 1 µg/L. The most abundant congeners of microcystin were MC-LR, -RR and YR.

**Figure 3:**
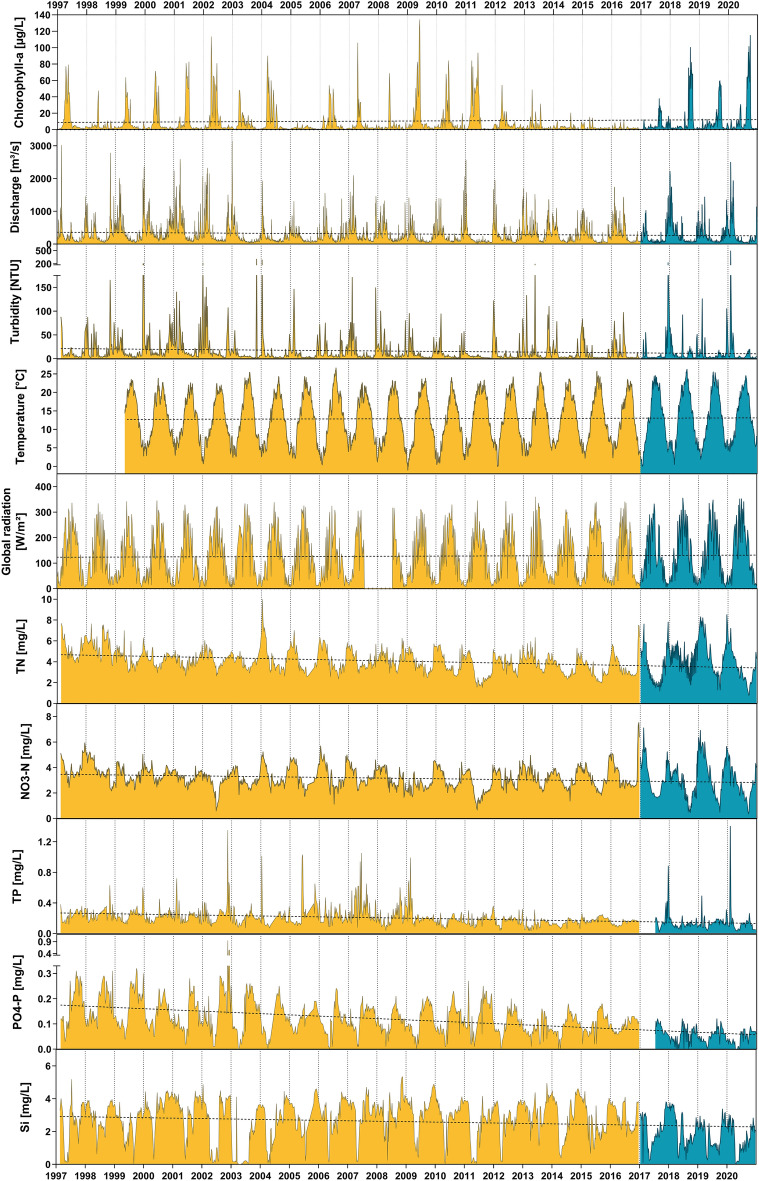
Long-term data of selected physico-chemical parameters of the Moselle in the 20-year reference period 1997–2016 (yellow) and in the period with cyanobacteria blooms 2017–2020 (blue-green). Discharge, global radiation, and water temperature are based on daily means, all other data (turbidity, chl-a, and nutrients) are based on weekly or bi-weekly measurements. All water parameters except for discharge were measured at the surface. A simple linear regression was interpolated using GraphPad Prism (Version 8.4.3).

**Figure 4:**
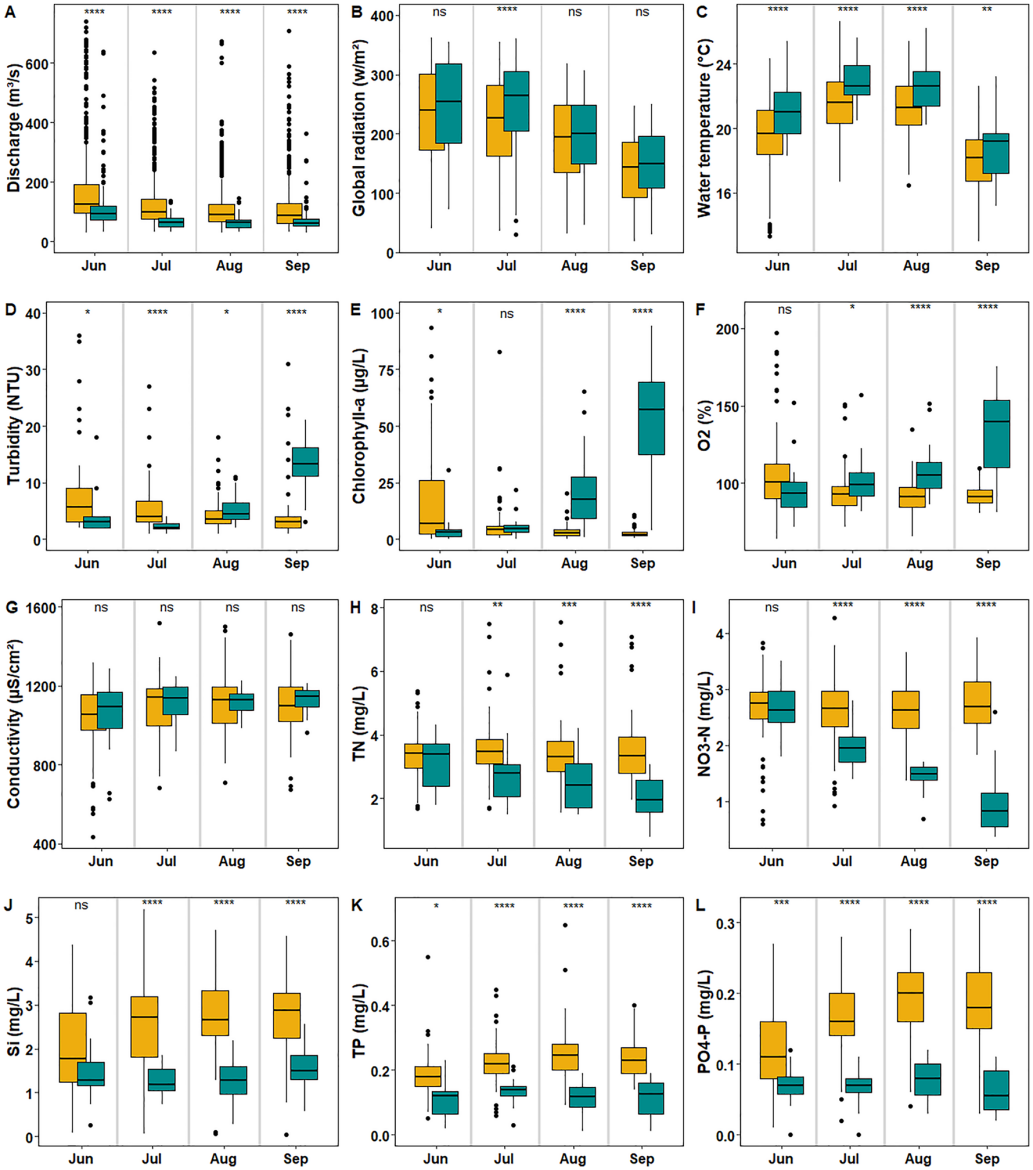
Boxplots (median, 25th and 75th percentiles, 1.5 × interquartile range) of selected environmental parameters for the months June/July/August/September in the 20-year reference period 1997–2016 (yellow) and in the period with cyanobacteria blooms 2017–2020 (blue-green). Values of continuous data (discharge, global radiation, conductivity and water temperature) are based on daily means, discontinuous data (turbidity, chl-a, oxygen (O_2_) and nutrients) are based on weekly means to avoid bias by higher sampling frequency during cyanobacteria blooms. All water parameters except for discharge were measured at the surface. Significant differences between periods were calculated by an ANOVA for each variable (*p < 0.05, **p < 0.01, ***p < 0.001, ****p < 0.0001). For better resolution, individual values of the period 1997–2016 are out of the y-axis limits: Discharge—13 values out of upper limits in June; turbidity—three values out of upper limits in June, one in July and one in September; chl-a–one value above upper limits in June; TP–three above limits in June.

A long-term dataset on physico-chemical parameters was analyzed to test which environmental conditions have triggered the occurrence of the cyanobacterial bloom (Figs. 3, S4). In a more detailed view we focused on the summer months of June, July, August and September, since the blooms developed and persisted during these months and compared the cyanobacterial bloom period from 2017 to 2020 with the previous 20-years as reference period (Fig. 4).

Discharge has decreased significantly since 1997 (Fig. 3, simple linear regression, *P* < 0.0001, and Fig. 4A, ANOVA, *P* < 0.0001, also compare Fig. S4). Discharge from June to September 2017–2020 only ranged between 69 and 76% of its previous 20-years median (Fig. 4A). The warm and dry weather in the summers of 2017–2020 was also reflected in a higher light availability with significantly elevated global radiation in July but not in the other months (Fig. 4B, ANOVA t, *P* < 0.0001). Albeit water temperature did not show an overall increase since 1997 (Fig. 3), it was significantly elevated by 0.9–1.8 °C in all summer months of the cyano-period compared to the previous 20-years median (Fig. 4C, ANOVA, *P* < 0.01). The temperature increase was particularly pronounced in June, July and August (*P* < 0.0001).

Turbidity decreased significantly in June and July compared to the reference period (Fig. 4D, 2-way ANOVA and Sidak’s post-test, *P* < 0.05), reflecting an overall decreasing trend (Fig. 3). In August and September, turbidity was significantly increased (*P* < 0.05), indicating a turbidity signal caused by the cyanobacteria bloom. The intensity of the latter was reflected in significantly elevated chl-*a* levels in August and September to 17.7 and 57.4 µg/L compared to the reference period (2.7 and 2.1 µg/L, Fig. 4E, ANOVA, *P* < 0.0001). In June median chl-*a* was significantly (ANOVA, *P* < 0.05) lower (3.0 µg/L) in 2017–2020 or in July equaled those in the reference period (7.1 µg/L). Cyanobacterial chl-*a* peaks since 2017 have counteracted the overall decreasing trend in chl-*a* concentrations since 1997 (Fig. 3). In accordance with cyanobacterial abundance, oxygen saturation significantly increased in July, August and September in the cyano-period (Fig. 4F, ANOVA, *P* < 0.05). Conductivity did not differ between the two periods in the Moselle (Fig. 4G).

All analyzed nutrients (silicate, TN, nitrate, TP, and phosphate) showed a significant decrease since 1997 (Fig. 3, simple linear regression, *P* < 0.001). In accordance with that all nutrients were significantly lower in the months July, August and September of the years 2017–2020 compared to the reference period (Fig. 4H–L, ANOVA, *P* < 0.01). With the establishment of the cyanobacteria bloom in August and September, this offset became more pronounced especially for dissolved P (PO_4_–P) and N (NO_3_–N).

**Figure 5:**
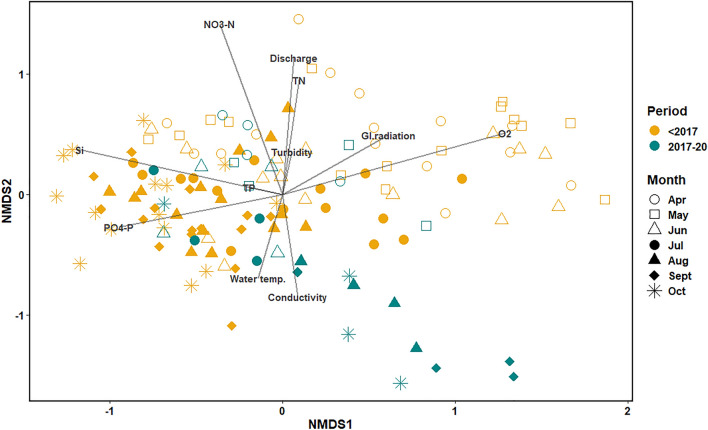
Phytoplankton community composition (monthly mean) based on biovolume of phytoplankton genera from microscopic counts displayed as NMDS graph and overlaid by environmental parameters. Each symbol represents the respective phytoplankton community composition in a given month and period (yellow–before cyanobacterial blooms, blue-green–with cyanobacterial blooms). Environmental parameters are monthly means based on weekly means. Years 2008–11 and 2013 were excluded from the analysis (see Materials and methods section).

To further analyze the phytoplankton community composition of the Moselle and correlating environmental factors we conducted a NMDS-analysis based on Bray–Curtis (phytoplankton community) and Euclidian (environmental factors) distance matrices (Fig. 5). The spring bloom community before 2017 (clear yellow circles, squares and triangles) clusters more to the upper right of the graph and correlates with high oxygen, high global radiation as well as low silica and low phosphate. The spring blooms in this period were dominated by diatoms of the genera *Skeletonema*, *Melosira*, *Stephanodiscus* and the broader group of “Centrales” (Fig. S2). The summer phytoplankton community composition before 2017 (filled yellow circles, triangles and diamonds) is clustered to the center left of the graph and is flanked by October samples. The total phytoplankton biomass in this season was much lower than in spring and dominant taxa were the cryptophytes *Rhodomonas* and *Cryptomonas*, next to diatoms and chlorophytes (compare Figs. 1, 2 and S2).

While the spring phytoplankton community in cyanobacteria years (clear blue-green circles, squares and triangles) seems to be similar to the spring/summer community in previous years, the summer community composition (filled blue-green circles, triangles and diamonds) was distinctly different and stretched out to the lower right of the graph. Those samples were dominated with up to 94% by *Microcystis aeruginosa* (individual data not shown, but compare Figs. 2 and S2). Low nitrogen (TN and nitrate), low discharge in combination with high water temperature and conductivity correlated best with the cyanobacteria-dominated samples in the 2017–20 period. A Mantel Test confirmed that physico-chemical parameters and phytoplankton community composition correlated significantly (*P* = 0.009). The correlation was less strong (*P* = 0.042) when discharge was excluded from the analysis.

Further statistical analysis showed that, the two periods (before and after 2017) differed significantly (ANOSIM) in their phytoplankton community composition. In more detail, phytoplankton genera that significantly correlated with the cyanobacteria period^20,21^ included the dominant genus *Microcystis*, but also other cyanobacteria e.g. *Dolichospermum, Pseudanabaena*, chlorophytes, e.g. *Desmodesmus,* and other taxa (Table S3). The reference period correlated best with diatoms such as the pennales group and *Stephanodiscus* but also *Cryptomonas*, chrysophyceae and chlorophytes (e.g. *Monorhaphidium*).

## Discussion

In this study we evaluated a long-term dataset for environmental conditions that caused cyanobacteria bloom formation in a regulated temperate river, the Moselle. During the past decades (since 1997), no cyanobacteria had been observed in the Moselle in high abundances, so that the occurrence of cyanobacterial blooms in 2017 and their regular recurrence since was unexpected. Albeit low cyanobacterial toxin concentration (i.e. microcystin) was suggesting no direct threat to humans^1^, variability was high and toxin concentrations in surface scums (not measured in this study) may be more problematic especially for children or dogs.

The analysis of long-term environmental conditions suggests that, cyanobacteria blooms were promoted by hydrological and physical conditions caused by extended dry and warm weather periods under non-limiting nutrient conditions. Dry and warm summers in recent years resulted in higher air temperatures and less precipitation and thus in a generally lower discharge in many rivers in Germany^12^. In the Moselle, water levels are stabilized by impoundments. Therefore, a decreasing discharge is not reflected in a low water level, but in reduced flow velocity and increased residence times in each of the impoundments. Reduced flow and higher residence time may give slow-growing cyanobacteria more time to grow and reach a critical mass for bloom development. In the Lower Darling River in Australia, flow velocities below 0.03 m/s were observed to promote *Microcystis* blooms^8^. In one impoundment of the Moselle, flow velocity was calculated to be around 0.7 m/s at mean flow conditions but as low as 0.15 m/s at low flow conditions (Federal Institute of Hydrology (BfG), unpublished data). Further studies need to clarify how flow velocities and residence times have changed in the Moselle in the recent years.

Next to discharge, water temperature seemed to be an important factor for the observed shifts in the phytoplankton community. In the Moselle, water temperature was significantly higher between June and September in the cyanobacteria period compared to the 20 years before. Temperature, next to flow velocity and residence time, was the most important driver for cyanobacterial biomass in regulated rivers in South Korea^16^. Warm water temperature results from higher air temperature and higher global radiation, but can also be promoted by low flow velocity and longer residence times as the relative input of solar energy is increased. *Microcystis* has a relatively low growth rate^22^ with a temperature optimum at relatively higher temperatures compared to other phytoplankton species. It thus has a growth advantage at higher temperatures^6^ and grows stronger in warmer months and normally forms blooms in summer. In addition, low discharge and high temperature may cause a thermal stratification which favors cyanobacteria that can regulate their buoyancy and thus their position in a stratified water column^4,23^.

Light availability is another important factor for phytoplankton growth. At the Moselle, global radiation significantly increased in July during cyanobacteria years, where together with warmer water temperature, a higher light availability is likely supporting *Microcystis* to reach higher biomass. Lower turbidity in July, likely caused by less precipitation and therefore less surface run-off and discharge may have further increased light availability in the water column. In the case of a large *Microcystis* bloom in the Lower Darling River in Australia, turbidity had the greatest influence on phytoplankton community composition, followed by discharge, water temperature, inorganic phosphorus and nitrogen^8^. However, turbidity did not significantly correlate with phytoplankton community in our study. This may be due to the fact that turbidity was generally very low in the Moselle and phytoplankton development itself affected turbidity.

In contrast to changes in discharge, temperature and light availability, nutrient concentrations were not significantly elevated in cyanobacteria years and therefore were not the primary trigger for the cyanobacterial bloom. As in many European catchments, phosphate and nitrogen were decreasing in the long-term trends due to improved management, while the increasing offset between dissolved nitrate and phosphate concentrations during the cyano-period were likely caused by nutrient uptake of the blooming cyanobacteria. Lower dissolved concentrations of silicate throughout the years after 2017 may be attributed to a lower turbidity and to lower inflow from surface run-off in the catchment in dry years. Nevertheless, while nutrient concentrations (nitrogen, phosphorus and silica) were declining over the study period, background nutrient levels remained sufficiently high to support high phytoplankton biomass^24^. Reduced flow and longer residence time may have enhanced the cyanobacteria’s capacity to use those nutrients. For the same reason, nutrient decrease did not seem to cause the decline of diatom abundance in spring since 2012. The cyano-period showed a short duration of limiting Si-conditions during spring, probably during Si-uptake by diatoms in the upper catchment. However, this period was short and similarly low values were visible in the range of the reference period.

Physical parameters such as water temperature or mixing, but also biological factors might explain the decrease of the diatom spring bloom since 2012. A likely factor is the invasion of the quagga mussel (*Dreissena rostriformis bugensis*) in the Moselle in 2011^19^, which may have increased the feeding pressure on diatoms. Quagga mussels are able to grow at relatively low temperatures and are therefore able to feed in the early growing seasons^25^, enhancing the pressure on the spring bloom. The effect of quagga mussels on cyanobacteria is still up for debate^25^, with some studies suggesting a positive effect on *Microcystis* dominance through selective filter feeding^26,27^ and others showing their suppression^28^.

In summary, our results suggest that with extended dry summer periods, rivers and especially impounded rivers will become more susceptible to harmful algae blooms due to reduced discharge, temperature rise and high light availability combined with sufficiently high nutrient supply. It remains unclear why other regulated rivers in Germany under similar hydrological conditions are so far not affected by cyanobacterial blooms. However, once established, *Microcystis* blooms may now easier recruit from benthic colonies remaining in low-flow zones of the river over the winter season^29^. It has been proposed that low redox situations in the sediment due to nutrient loading and eutrophication may support *Microcystis* bloom formation in lakes^30^. Albeit we did not observe low oxygen situations in the lower section of the river, such conditions with consequences for summer phytoplankton blooms were found in an upstream tributary^31^. Measures to further decrease nutrient (N and especially P) levels may help to limit cyanobacteria blooms in the Moselle, since currently they are at a non-limiting level for phytoplankton growth. Other measures to mitigate cyanobacteria blooms in rivers are limited. There have been suggestions to counteract cyanobacteria blooms in rivers by manipulation of discharge^32^, like pulsed flow^18,33^, flushing from upstream storage^34^, or limitation of water extraction^35^. Especially at the onset of a bloom in late July or early August this may be a suitable management tool in the Moselle. However, some of these measures require sufficient water availability upstream, which is not always the case, especially during drought periods and may cause conflicting interests. e.g. with river navigation. Moreover, this approach is not always leading to the intended effects. In a model of the Geum River (South Korea), the open-gate scenario did improve the situation upstream, but predicted more blooms downstream^18^.

On a global scale, many rivers are regulated^13^ and despite accomplished measures and improvements in water quality, eutrophication remains one of the main challenges for water quality^36^. Nevertheless, river phytoplankton is mostly dominated by diatoms and/or green algae but not by cyanobacteria^24^. The occurrence of cyanobacteria in the Moselle therefore was unexpected, but can be explained by the processes linked to climate change as described above. This event shows that with the effects of climate change getting more extreme, European rivers will face unprecedented developments in the near future. In this regard, the Moselle may serve as an example for the future of regulated temperate rivers.

## Methods

### Sampling and field measurement

The Moselle River is located in the low mountain ranges of western Europe, between France, Luxemburg and Germany (Fig. S1). Its water quality is monitored by several authorities on a regular basis. Samples for the present study were taken from pontoons at the Moselle in Koblenz, 2 km (1997) and 6 km (from 1998) upstream of the confluence with the larger river Rhine. The data are therefore representative only for the lower section of the Moselle. The monitoring has been maintained in the same or similar way by the Federal Institute of Hydrology since the early 1980s. Due to data quality and in order to avoid bias by the eutrophication-period until the early 1990s, we here include only data since 1997. To our knowledge, cyanobacteria blooms have not occurred in the lower Moselle before the analyzed period. Samples were taken on a weekly basis. During the periods of cyanobacterial blooms, the sampling interval was increased to two or three samples per week. Continuous data of discharge (from 1997 to 2020) and water temperature (from 1999 to 2020) at the station in Cochem, 45 km upstream of the sampling station, were measured and provided by the Federal Waterways and Shipping Agency (WSV). Data for global radiation were used from the German Weather Service station Trier-Petrisberg (No. 05100) at river km 193 (roughly 95 km from the water quality sampling station).

Oxygen concentration and saturation were determined directly in the field by a portable probe (Hach HQ 30d flexi, Hach Loveland, Colorado, USA). About 15 L of water were sampled as a grab sample with a bucket from the surface from a pontoon that reached about 20 m into the river. The sampling of cyanobacterial scums occasionally forming at the river banks was avoided or scums were destroyed by moving the bucket several times up and down on the surface. The sampled water was stored in a plastic container and transported to the laboratory for further analyses. For biweekly phytoplankton analyses, a subsample of 250 ml or 100 ml was transferred in brown glass bottles and conserved with Lugol’s iodine solution (0.5% final concentration) directly in the field.

### Laboratory analysis

The water sample was processed within 5 h after collection as follows: Conductivity was measured by a probe (WTW conductometer LF191 / LS1/T-1,5 Xylem analytics, Langenhagen, Germany). Turbidity was measured by a 2100P turbidimeter (Hach Lange, Düsseldorf, Germany). Subsamples for dissolved nutrients were filtered (Whatman PURADISC AQUA 30/0.45 Cellulose-acetate), and stored in sterile 15 ml centrifuge tubes (PP, ROTILABO®, Carl Roth, Germany) at − 20 °C until further analysis. Subsamples for TN and TP were fixed with sulfuric acid (0.33% final concentration).

Nutrients (TN, TP, NO_3_, PO_4_, Si) were analyzed in the laboratory either by colorimetrical cuvette assays (Hach Lange, Düsseldorf, Germany), or using the San^++^System (Skalar Breda Netherlands). After 2017 TN and TP were measured by an external company (Institut Dr. Nowak, Ottersberg Germany) according to EN 12,260-H34:2003-12 and ISO 6878-D11:2004-09.

For chlorophyll and toxin analysis, between 0.5 and 1.5 L of water were filtered on a glass fiber filter using a vacuum pump. Filters for toxin analysis were stored at − 20 °C until further analysis. Chlorophyll-*a* content was determined photometrically after immediate hot ethanol extraction after DIN 38,409–60. Since 2004, chlorophyll-fluorescence was additionally determined using the AlgaeLabAnalyzer (bbe Moldaenke GmbH, Schwentinental, Germany) allowing the discrimination of the four algae groups: chlorophytes, cyanobacteria, chrysophytes and diatoms^37^. Detailed phytoplankton community composition was determined based on microscopic taxonomical evaluation by external companies according to standard procedures^38^.

### Screening for cyanobacterial toxins

In order to extract intracellular toxins, 0.5–1 L of surface water was filtered through a glass fiber filter (Whatman GF 6, Ø 55 mm, Dassel, Germany). The filters with the residue were transferred into 15 ml centrifuge tubes (PP, ROTILABO®, Carl Roth, Germany) and stored in a freezer at − 25 °C for a minimum of 24 h. After that, 5 ml methanol (LC/MS grade, Merck Chemicals, Germany) was added to the residue, sonicated for 10 min (RK 102H BANDELIN electronic, Germany) and centrifuged at 4000 rpm (Hettich Rotina 380 R, Andreas Hettich GmbH & Co. KG, Germany). An aliquot of 1 ml extract was transferred into a 15 ml centrifuge tube, spiked with 10 µl internal standard solution (1 ng/µl, Table S1) and evaporated with nitrogen at 45 °C (TurboVap® LV, Biotage, Sweden). The residue was reconstituted in 1 mL supra pure water/methanol (90:10), homogenized, filtered using a membrane syringe filter (Spartan 13/0,45 RC, Whatman, Dassel, Germany) and finally transferred into a glass HPLC vial. The extract was stored at 8 °C until LC–MS/MS measurement.

LC-MS/MS analysis of Microcystins (MC) in sample extracts were carried out using a SCIEX ESI Triple Quad 6500 + mass spectrometer (AB Sciex Germany GmbH, Germany) coupled to an Agilent 1200 series HPLC system (Agilent Technologies Deutschland GmbH, Germany) consisting of high performance autosampler (HiP ALS, G1367B), thermostat for autosampler (1290 Infinity, Termostat, G1330B), binary pump (Bin Pump SL, G1312B) and thermostatted column compartment. Chromatographic separation of the analytes was achieved by an Agilent eclipse plus C18 column (150 mm × 2.1 mm, 3,5 µm) and mobile phases consisting of 0.1% formic acid in supra pure water (eluent A) and acetonitrile (eluent B). The gradient steps for Eluent A were: 0–1 min 100%, 2 min 80%, 16.5 – 19 min 0%, 19.1–25 min 100%. The flowrate was set to 0.3 ml/min, column temperature was set to 40 °C and injection volume to 80 µl. All used solvents were LC/MS grade (Merck Chemicals, Germany). MC standards (Table S1) were purchased form Cyano Biotech, (Berlin, Germany) and internal standards (IS) were purchased from Cambridge Isotopes (Tewksbury, MA, USA). Calibration samples were prepared in ultra-pure water/methanol (90:10), a MC concentration range of 0.01–100 µg/L and an IS concentration of 10 µg/L. Measured concentrations below quantification limits were noted as half of the respective quantification limit. Further information about LC/MS conditions can be found in the SI (Table S2).

### Statistical analysis

All data and statistical analyses were performed with R version 4.2.3 and RStudio 2023.03, if not stated differently. Continuous data were available as daily means (discharge, global radiation and water temperature). All other environmental parameters were summarized as weekly means in order to avoid biases by shorter sampling intervals during the cyanobacterial growth season. From these daily or weekly means, monthly medians were calculated for each time period: the reference period before the occurrence of cyanobacteria in the Moselle 1997–2016 and the period between 2017 and 2020 when cyanobacteria blooms were observed in the Moselle. Significant differences between the two periods were calculated per month using an ANOVA within the ‘stat_compare_mean’ function in the R ggplot package (version 3.4.2) and significance levels of *p < 0.05, **p < 0.01, and ***p < 0.001.

To analyze changes in phytoplankton community composition and abundance, we used the biovolume as determined from microscopic analyses. Biovolume was calculated according to Mischke & Behrendt (2015). To eliminate biases potentially introduced by different taxonomists during the 24-year period, we evaluated the data on the phylogenetic level of the genus. Nevertheless, due to strong biases attributable to different taxonomists we had to remove the phytoplankton data of the years 2008–2011 and 2013 from the analysis. Monthly averages of phytoplankton taxa biovolume were calculated for the growth season between April and October. Non-metric multi-dimensional scaling (nmds) was performed based on these data and overlaid with environmental parameters. An analysis of similarities (ANOSIM) tested for significant differences in the phytoplankton community between the two periods (1997–2016 and 2017–2020). The ‘multipatt’ function in R (package *indicspecies*, v. 1.7.13) analyses the association of indicator species and site groups^20,21^. Its application returned genera that were significantly correlated to each period. Significant correlations of individual environmental parameters and the phytoplankton dataset were returned by the ‘envfit’ function in R (package *vegan*, v. 2.6–4). A mantel test based on Pearson's product-moment correlation (999 permutations) was performed on a Bray–Curtis distance matrix of phytoplankton data and on a Euclidian distance matrix of environmental data.

### Supplementary Information

Supplementary Information.

## Data Availability

All data can be downloaded on https://iksr.bafg.de/iksr/ or can be provided up on reasonable request (phytoplankton data). Data on global radiation can be downloaded from the German Weather Service and data on discharge can be downloaded from the Federal Waterways and Shipping Agency (WSV).
