# Supplementary material for: Climate change triggered planktonic cyanobacterial blooms in a regulated temperate river
Source: Sci Rep. 2024 Jul 15;14:16298. doi: 10.1038/s41598-024-66586-w (PMC11250786; doi:10.1038/s41598-024-66586-w)
Supplement: Supplementary file 1 — Supplementary Information. [file 41598_2024_66586_MOESM1_ESM.docx]

Supplemental Material

**Climate change triggered planktonic cyanobacterial blooms
in a regulated temperate river**

Authors:

**Julia Kleinteich*, Marieke A. Frassl, Manoj Schulz, Helmut Fischer**

Federal Institute of Hydrology (BfG), Germany

Am Mainzer Tor 1, 56068 Koblenz, Germany

[Kleinteich@bafg.de](mailto:Kleinteich@bafg.de), [Frassl@bafg.de](mailto:Frassl@bafg.de), [Manoj.Schulz@bafg.de](mailto:Manoj.Schulz@bafg.de), [Helmut.Fischer@bafg.de](mailto:Helmut.Fischer@bafg.de)

*corresponding author

**Table S1: Analytical Standards**

| **Analytical Standards** | **CAS** | **Catalogue No.** | **Vendor** |
| --- | --- | --- | --- |
| Microcystin-RR | 111755-37-4 | MCRR-a | Cyano Biotech GmbH, Berlin, Germany |
| [D-Asp3,(E)-Dhb7]-Microcystin RR | 202120-08-9 | MCADRR-a | Cyano Biotech GmbH, Berlin, Germany |
| Microcystin-LA | 96180-79-9 | ULM-10346-1.2 | Cambridge Isotopes, Tewksbury, USA |
| Microcystin-LF | 154037-70-4 | MCLF-a | Cyano Biotech GmbH, Berlin, Germany |
| Microcystin-LR | 101043-37-2 | MCLR-a | Cyano Biotech GmbH, Berlin, Germany |
| [D-Asp3]-Microcystin-LR | - | ALX-350-173-C025 | Enzo Life Sciences GmbH, Lörrach, Germany |
| Microcystin-LW | 157622-02-1 | MCLW-a | Cyano Biotech GmbH, Berlin, Germany |
| Microcystin-LY | 123304-10-9 | MCLY-a | Cyano Biotech GmbH, Berlin, Germany |
| Microcystin-WR | 138234-58-9 | ALX-350-167-C025 | Enzo Life Sciences GmbH, Lörrach, Germany |
| Microcystin-YR | 101064-48-6 | MCYR-a | Cyano Biotech GmbH, Berlin, Germany |
| Microcystin-HtyR | - | ALX-350-174-C025 | Enzo Life Sciences GmbH, Lörrach, Germany |
| Nodularin | 118399-22-7 | NOD-a | Cyano Biotech GmbH, Berlin, Germany |
| Cylindrospermopsin | 143545-90-8 | CYN-a | Cyano Biotech GmbH, Berlin, Germany |
| 15N13 Microcystin-RR | 111755-37-4 (unlabeled) | NLM-10340-1.2 | Cambridge Isotopes, Tewksbury, USA |
| 15N10 Microcystin-YR | 101064-48-6 (unlabeled) | NLM-10343-1.2 | Cambridge Isotopes, Tewksbury, USA |
| 15N7 Microcystin-LA | 96180-79-9 (unlabeled) | NLM-10345-1.2 | Cambridge Isotopes, Tewksbury, USA |
| 15N10 Microcystin-LR | 101043-37-2 (unlabeled) | NLM-10295-1.2 | Cambridge Isotopes, Tewksbury, USA |

**Table S2: LC-MS/MS analytes and m/z ratios of detected ions.**

| **Analyt** | **Precursor ion (m/z)** | **Product ion (m/z)** | **DP (V)** | **CE (V)** | **CXP (V)** |
| --- | --- | --- | --- | --- | --- |
| Cylindrospermopsin | 415,9 | 336,0 | 81 | 29 | 10 |
|  |  | 194,2 |  | 51 | 14 |
| Nodularin | 825,4 | 162,9 | 100 | 50 | 10 |
|  |  | 69,9 |  | 107 | 6 |
| Microcystin-LA | 910,5 | 135,1 | 131 | 77 | 10 |
|  |  | 107,2 |  | 107 | 8 |
| Microcystin-LF | 986,6 | 135,0 | 91 | 93 | 14 |
|  |  | 107,1 |  | 107 | 8 |
| Microcystin-LR | 498,3 | 135,1 | 90 | 40 | 10 |
|  |  | 103,1 |  | 90 | 10 |
| [D-Asp3]-Microcystin-LR | 981,5 | 103,0 | 171 | 171 | 12 |
|  |  | 91,0 |  | 179 | 10 |
| Microcystin-LW | 1025,6 | 213,4 | 101 | 61 | 6 |
|  |  | 135,1 |  | 91 | 12 |
| Microcystin-LY | 1002,5 | 265,2 | 121 | 61 | 8 |
|  | 501,8 | 135,1 | 90 | 40 | 10 |
| Microcystin-RR | 519,9 | 103,1 | 91 | 89 | 8 |
|  |  | 135,0 |  | 43 | 10 |
| [D-Asp3,(E)-Dhb7]-Microcystin-RR | 512,8 | 135,0 | 91 | 35 | 12 |
|  |  | 103,0 |  | 91 | 12 |
| Microcystin-WR | 1068,5 | 1040,4 | 211 | 61 | 18 |
|  |  | 103,1 |  | 175 | 10 |
| Microcystin-YR | 523,3 | 135,1 | 90 | 40 | 10 |
|  |  | 103,1 |  | 90 | 10 |
| Microcystin-HtyR | 530,4 | 135,0 | 51 | 15 | 12 |
|  |  | 107,0 |  | 87 | 16 |
| 15N13 Microcystin-RR | 526,3 | 135,0 | 56 | 35 | 8 |
|  |  | 103,0 |  | 91 | 14 |
| 15N10 Microcystin-LR | 503,3 | 103,1 | 25 | 79 | 12 |
|  |  | 135,0 |  | 15 | 18 |
| 15N10 Microcystin-YR | 528,3 | 103,1 | 30 | 83 | 14 |
|  |  | 135,0 |  | 15 | 8 |

**Table S3**: Multilevel pattern analysis (multipatt function in R package indicspecies version 1.7.13) of the phytoplankton community composition (genus level) comparing the reference period (1997-2016) with the cyanobacteria period (2017-2020). Total number of taxa: 168. Selected number of taxa: 45. Only taxa that show a significant (p<0.05) association with one of the two groups (reference period and cyanobacterial period) are listed and sorted after the IndVal.g value that is a measure for the associations between a species and a group (for details see De Cáceres et al. (2010)*).

| **Associated taxa** | |
| --- | --- |
| **Reference period (1997-2016)** | **Cyanobacteria period (2017-2020)** |
| *Cryptomonas* | *Ochromonas* |
| *Pennales* | ***Microcystis*** |
| *Chrysophyceae* | *Actinocyclus* |
| *Asterionella* | *Spermatozopsis* |
| *Monorhaphidium* | *Tetrachlorella* |
| *Kephyrion / Pseudoskephyrion* | *Oocystis* |
| *Stephanodiscus* | *Pseudodictyosphaerium* |
| *Chlorococcales* | *Pseudanabaena* |
|  | *Ulnaria* |
|  | *Aphanocapsa* |
|  | *Eutetramorus* |
|  | *Pseudopedinella* |
|  | *Chroococcus* |
|  | *Granulocystis* |
|  | *Crucigeniella* |
|  | *Chrysochromulina* |
|  | *Coenochloris* |
|  | *Desmodesmus* |
|  | *Pseudopediastrum* |
|  | *Peridiniopsis* |
|  | *Nephrochlamys* |
|  | *Plagioselmis* |
|  | *Staurosira* |
|  | *Trachydiscus* |
|  | *Katablepharis* |
|  | *Anathece* |
|  | *Cyanodictyon* |
|  | *Mychonastes* |
|  | *Polytoma* |
|  | *Ulotrichale* |
|  | *Dolichospermum* |
|  | *Achnanthes* |
|  | *Ankyra* |
|  | *Dinophyceae* |
|  | *Stauroneis* |
|  | *Provasoliella* |
|  | *Thorakochloris* |

* De Cáceres M., Legendre P. and Moret M.: Improving indicator species analysis by combining groups of sites. Oikos 119: 1674-1684, 2010. doi: 10.1111/j.1600-0706.2010.18334.x


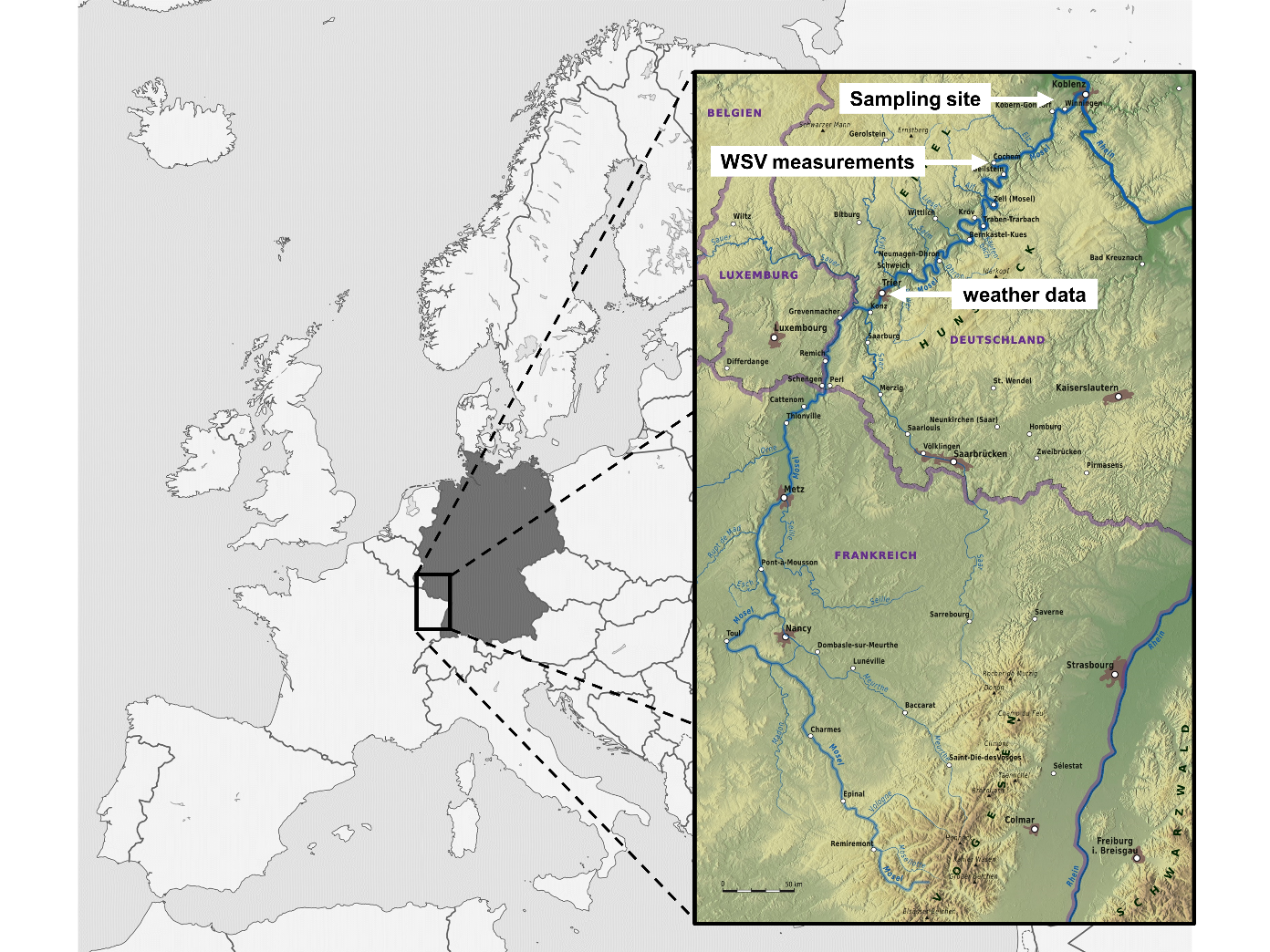


Figure S1: Map of the Moselle and sampling site at Coblenz, measurement station of continuous discharge and temperature data in Cochem by the shipping authority (WSV), and location of the weather station in Trier, Germany. Right map based on openstreetmaps and modified by TomGonzales and Gete1.


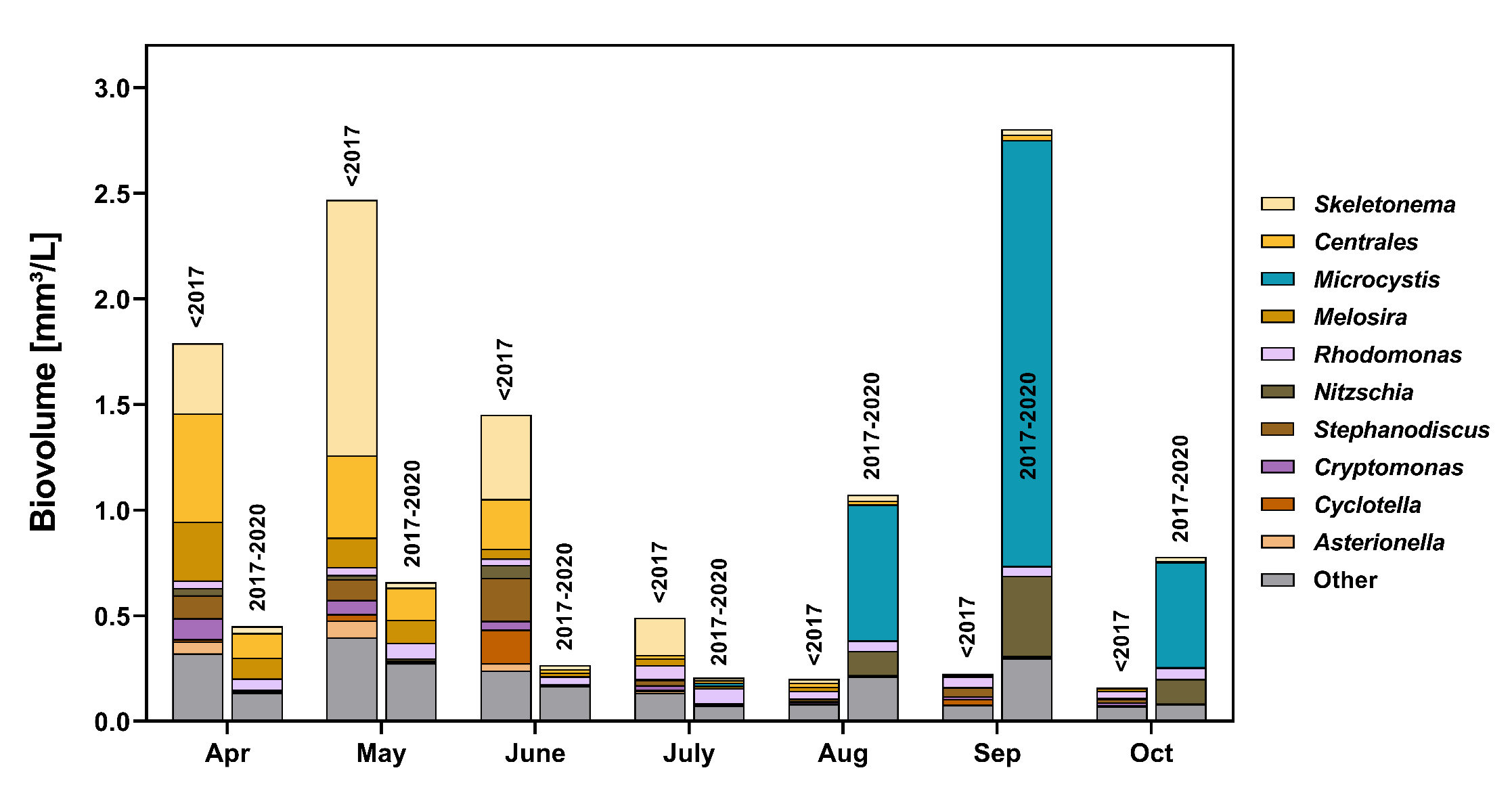


**Figure S2**: Seasonal phytoplankton abundance of the top ten most abundant genera, based on average biovolume from microscope counting (see material and methods section for details). Genera are sorted in decreasing abundance. Groups were separated into the years before 2017 and 2017-2020. Diatom genera are displayed in yellow/brown colors, cryptophytes in purple and cyanobacteria i.e. *Microcystis* as the sole genus of this group in the top ten taxa in blue-green.


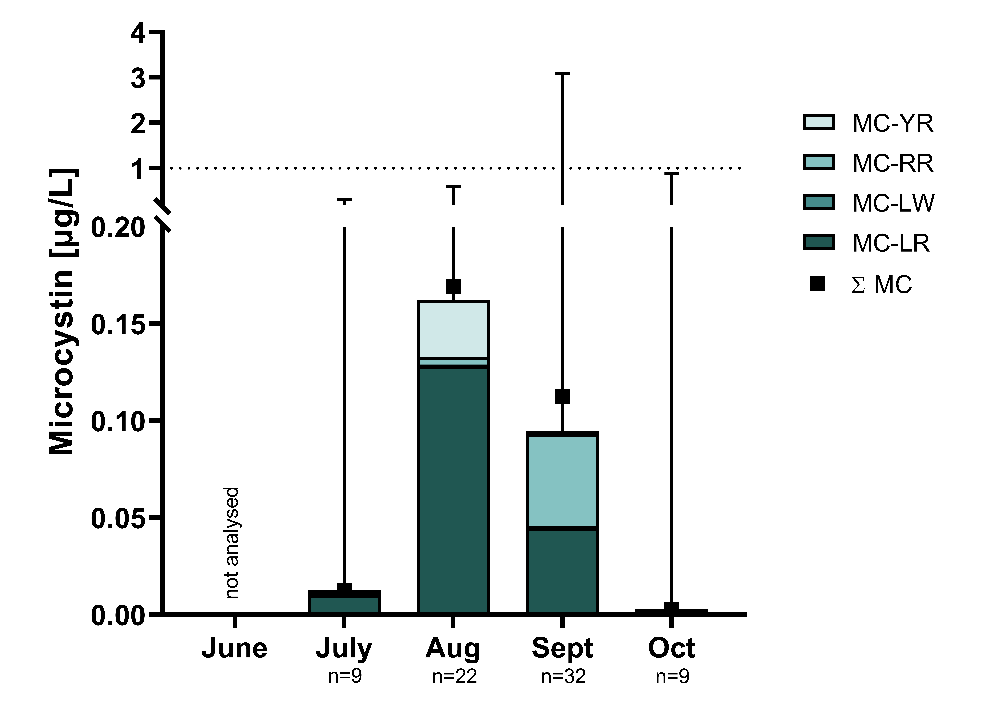


**Figure S3**: Microcystin concentrations in the Moselle at Koblenz (2017-2020). Medians of all congeners (MC-YR, MC-RR, MC-LW and MC-LR) and the median of all detectable MCs with range (Min-Max) are shown as derived based on LC-MS/MS analysis. The dashed line indicates the WHO drinking water guideline value (the value for recreational waters is 24 µg/L). Concentrations below quantification limits were noted as half of the respective quantification limit.


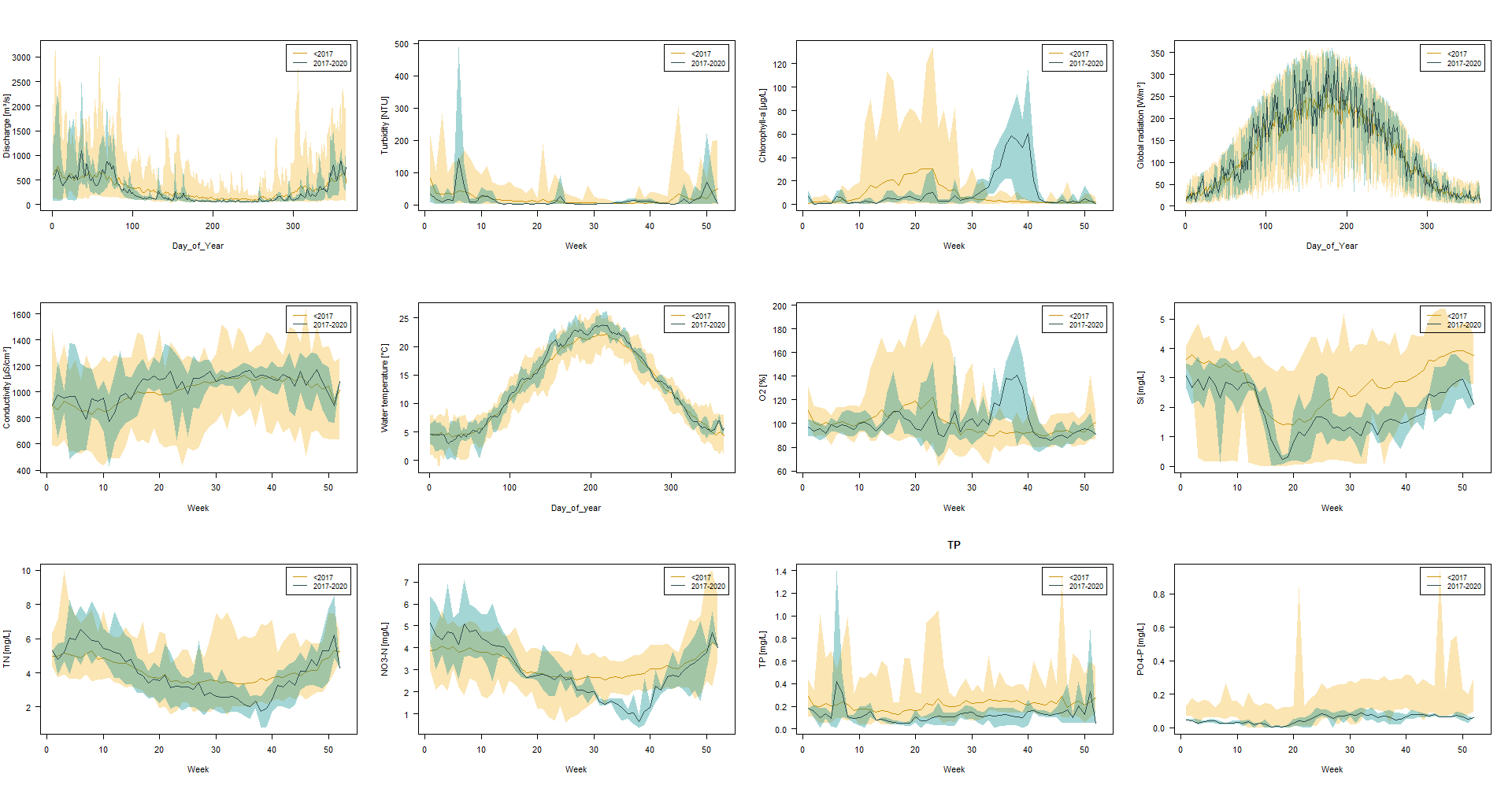


**Figure S4**: Complete seasonal cycles of environmental parameters in the Moselle at Koblenz based on daily (discharge, global radiation, conductivity and water temperature) or weekly averages for the reference period 1997-2016 (yellow) and the cyanobacteria period from 2017-2020 (blue).
